# Genetic variants of accessory proteins and G proteins in human genetic disease

**DOI:** 10.1080/10408363.2024.2431853

**Published:** 2025-01-01

**Authors:** Miles D. Thompson, Peter Chidiac, Pedro A. Jose, Alexander S. Hauser, Caroline M. Gorvin

**Affiliations:** a Krembil Brain Institute, Toronto Western Hospital, Toronto, Ontario, Canada; b Department of Physiology and Pharmacology, University of Western Ontario, London, Ontario, Canada; c Division of Renal Diseases & Hypertension, Departments of Medicine and Pharmacology/Physiology, The George Washington University School of Medicine and Health Sciences, Washington, District of Columbia, USA; d Department of Drug Design and Pharmacology, Faculty of Health and Medical Sciences, University of Copenhagen, Copenhagen, Denmark; e Institute of Metabolism and Systems Research (IMSR), University of Birmingham, Birmingham, West Midlands, UK

**Keywords:** G protein-coupled receptor (GPCR), accessory protein, G protein, pharmacogenetics, genetics

## Abstract

We present a series of three articles on the genetics and pharmacogenetics of G protein- coupled receptors (GPCR). In the first article, we discuss genetic variants of the G protein subunits and accessory proteins that are associated with human phenotypes; in the second article, we build upon this to discuss “G protein-coupled receptor (GPCR) gene variants and human genetic disease” and in the third article, we survey “G protein-coupled receptor pharmacogenomics”. In the present article, we review the processes of ligand binding, GPCR activation, inactivation, and receptor trafficking to the membrane in the context of human genetic disease resulting from pathogenic variants of accessory proteins and G proteins. Pathogenic variants of the genes encoding G protein α and β subunits are examined in diverse phenotypes. Variants in the genes encoding accessory proteins that modify or organize G protein coupling have been associated with disease; these include the contribution of variants of the regulator of G protein signaling (RGS) to hypertension; the role of variants of activator of G protein signaling type III in phenotypes such as hypoxia; the contribution of variation at the *RGS10* gene to short stature and immunological compromise; and the involvement of variants of G protein-coupled receptor kinases (GRKs), such as GRK4, in hypertension. Variation in genes that encode proteins involved in GPCR signaling are outlined in the context of the changes in structure and function that may be associated with human phenotypes.

## Introduction

In addition to the genes encoding heterotrimeric G proteins, those encoding the accessory proteins and effectors involved in signaling processes are often critical to G protein-coupled receptor (GPCR) signaling [1,2]. With the applications of whole exome sequencing (WES), whole genome sequencing (WGS), and RNA sequencing (RNASeq) to clinical diagnostics [3], variant interpretation of genes integral to GPCR signaling has become increasingly important. Here we will discuss the role of genetic variation in genes encoding heterotrimeric G proteins and accessory proteins that alter the GPCR signaling associated with human phenotypes.

**Figure 1.**
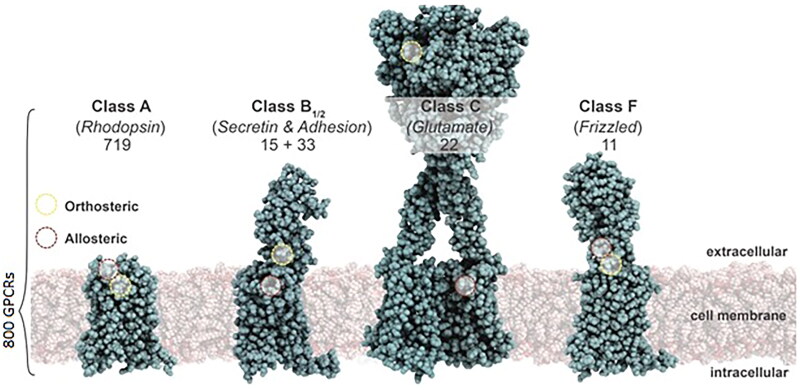
A depiction of the three major subclasses of GPCR. Class A receptors, or rhodopsin like receptors, share sequence similarity to rhodopsin and the calcitonin receptor and bind ligands within the transmembrane domain. Class B receptors, including secretin/adhesion receptors such as glucagon-like receptors, have large extracellular domains and bind ligands in multiple steps. Class C GPCRs such as the calcium sensing receptor (CaSR) and metabotropic glutamate receptors bind endogenous and allosteric ligands within their large extracellular domain. Class F receptors encode the smoothened receptor targeted by antineoplastic agents.

With respect to GPCR pharmacogenomics, there are four receptor subclasses: the class A receptors, characterized by transmembrane ligand binding sites that share sequence similarity with rhodopsin [1]; the class B receptors, which are subdivided into class B1 and the class B2 adhesion GPCRs; the class C receptors such as the metabotropic glutamate receptors and the calcium sensing receptors (CaSR), which bind extracellular endogenous and/or allosteric ligands in order to elicit conformational changes associated with signaling; and the class F receptors, which encode the smoothened receptor targeted by antineoplastic agents [1–4] (Figure 1).

## G protein-coupled receptors

We will discuss the contribution of genetic variants in accessory proteins and G proteins that impact the signaling of class A GPCRs, the most common class of GPCRs [3,4]. A brief summary of the features of GPCRs orients this discussion on how disruption of accessory proteins and G proteins result in disease.

**Figure 2.**
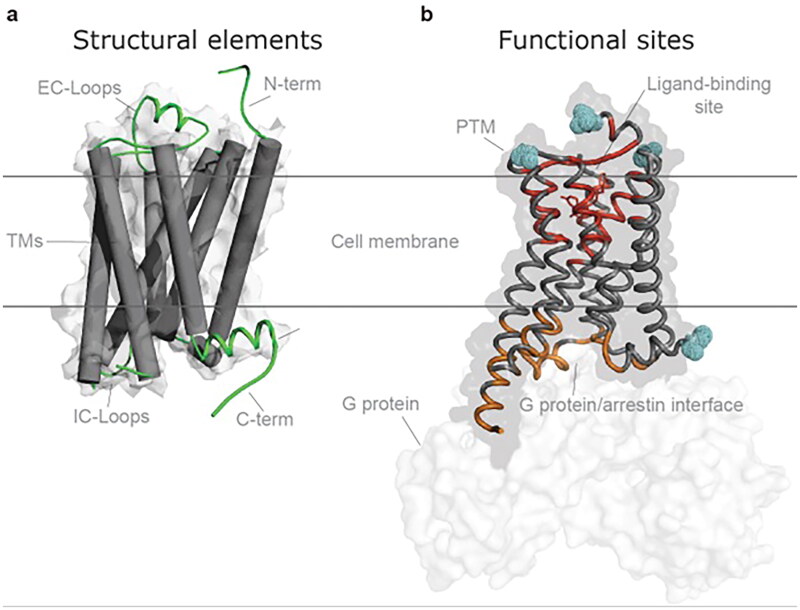
Schematic representation of GPCR structural and functional sites. **a**, the seven transmembrane helices (shown as tubes) spanning through the cell membrane phospholipid bilayer being connected by three extracellular loops (ECLs) and three intracellular loops (ICLs). Transmembrane residues are most conserved among GPCRs, whereas the loops and the C and N termini (intra- and extracellular, respectively) are highly flexible. Helix 8 often positions intracellularly parallel to the cell membrane. In TM3 (TM, transmembrane), a conserved cysteine (Cys3x25) often forms a cysteine-bridge (not shown) to ECL2 (Cys45x50). **b**, the orthosteric ligand-binding domain is extracellular (red) and the G protein/arrestin interface is at the intracellular side (orange). Posttranslational modifications (blue) including phosphorylation, glycosylation and ubiquitination add to the functional diversity of GPCRs.

The extracellular amino terminus of class A GPCRs is inserted through the plasma membrane. The proteins form seven transmembrane domains (TM), with three extracellular and an intracellular carboxyl terminus (Figure 2a) [1,2]. The functional structures of GPCRs include ligand binding-site(s), the motifs necessary for accessory proteins to respond to conformational changes specific to a ligand and facilitate selection of the effector [5–10].

Ligands, including peptides and proteins, tend to bind N-terminal extracellular loops (ECL) and TM domains (Figure 1). The ligands of many receptors, including dopamine, however, tend to bind to the hydrophobic α-helices [11,12]. While ligand specificity may be defined by multiple GPCR motifs [13], the binding pockets may have alternative binding domains or accommodate ligands in multiple orientations that introduce “signaling bias” [2].

The classification of pathogenic or likely pathogenic variants of G proteins and accessory proteins in disease is presented in the context of GPCR motifs critical to signaling [14]. We will review data on G protein structure and function before examining genotype-phenotype correlations[15–53].

### G protein coupling

Heterotrimeric G proteins consist of Gα-, Gβ-, and Gγ-subunits. Each Gα-subunit of each heterotrimer is bound to one guanosine 5′-diphosphate (GDP). The Gβγ subunits form a stable dimer. Once activated, a GPCR interacts with the G protein, a high-affinity complex consisting of receptor-G protein is formed [16]. The GDP is then released, and guanosine 5′-triphosphate (GTP) binds to Gα [51–53].

The process of G protein coupling involves several steps. Once ligand binds, the conformational ensemble in which the GPCR exists (which is a mixture of active and inactive conformations, with inactive conformations being predominant for most agonist-free receptors) is shifted toward more active conformations. While the activated receptor subsequently interacts with the three subunits (Gα-, Gβ-, and Gγ-subunits) of the inactive heterotrimeric G protein, there are still receptors in inactive conformations despite having bound the agonist. Crystal structures of agonist-bound receptors in the absence of G protein have shown that, in this state, the receptor does not exist in a fully active conformation: G protein interaction shifts the conformational ensemble even further toward active conformations.

**Figure 3.**
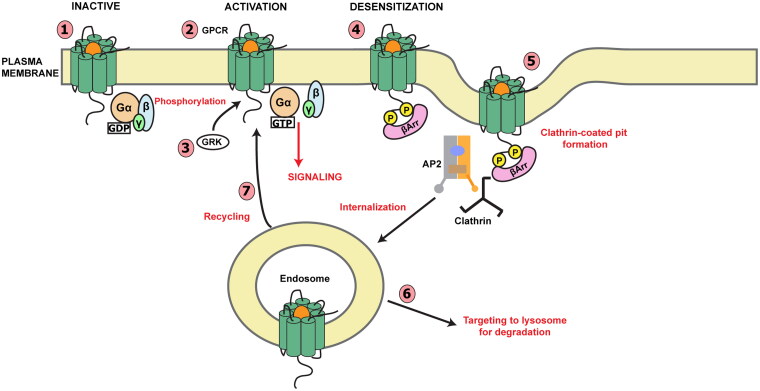
Schematic of G protein-coupled receptor (GPCR) activation and inactivation. (1) Agonist (orange) binds to the GPCR, initiating conformational changes in the receptor, resulting in exchange of GDP for GTP at the G protein α-subunit. (2) The G protein α and βγ subunits dissociate and signaling is initiated. (3) GRK is recruited, displacing enzyme and phosphorylating the agonist-occupied receptor. (4) β-arrestin (βarr) forms a complex with the receptor. Some GPCRs are able to initiate signaling pathways that involve βarr. (5) The receptor is internalized at clathrin-coated pits which requires the heterotetrameric adaptor protein 2 (AP2), βarr, and clathrin proteins. (6) Internalized receptor is directed to the endosome, from which it can be directed to the lysosome for degradation, or (7) dephosphorylated receptor may be recycled to the plasma membrane [54]. GDP guanosine 5′-diphosphate, GTP guanosine 5′-triphosphate.

This classic model outlined is a generalization from the data and has undergone revisions [51–53]. The GPCR–G protein effector complexes generate second messengers or operate ion channels that may be further regulated by auxiliary proteins [53]. Figure 3 outlines these processes schematically.

### GPCR variants

While not the emphasis of this text, naturally occurring or *in vitro* designed variants have been shown to disrupt GPCR activation pathways [47,48]. For example, a conservative leucine to isoleucine GPR35 substitution can change the Gα13 and Gα12 specificity [48]. Our understanding of GPCR-G protein activation mechanisms has grown as a result of improved computational methods and increasing number of structures reported for all classes of GPCRs [49]. By analyzing the structure of 45 GPCRs in active, inactive, and agonist/antagonist-bound states, common pathways of receptor activation and coupling to G proteins have been described [50].

Conformational changes that result in signaling of GPCR pathways can be modeled in four stages. To begin, there is a signal initiation step that is dependent on contacts between residues in ligand-binding and Na^+^ pockets. Next, the hydrophobic lock between residues of TM3 and TM6 is broken, allowing TM6 to move outward and facilitate the motion of TM7 toward TM3, resulting in changes within the conserved NPxxY motif. In the final step, the intrahelical contacts of the conserved R^3x50^ residue, and its ionic lock with D(E)^6 × 50^, are released, facilitating further outward movement of TM6 and allowing the G protein to bind on the cytosolic side [51]. Most structural investigations have been performed on class A receptors, and, while TM6 is a universal helix macro-switch, it differs mechanistically among classes on the residue level [35]. This shows that, despite sharing the same overall structural scaffold, individual GPCR classes differ in their microswitch residues, their location, and their side-chain interactions.

### GPCR co-coupling

Down-stream, multiple signaling pathways may be triggered by single receptor types, and other signaling cascades may also be activated by hetero- and homo-oligomerization of receptors that may be localized on lipid rafts [2,16]. The diversity of signaling from one receptor type in response to multiple ligands, each with their own “signaling bias”, putatively results from unique GPCR conformational changes. In turn, “biased signaling” may result in the selection of effectors due to the action of G proteins, GPCR kinases, arrestins, and possibly other accessory proteins [5].

An emerging “map” of GPCR co-coupling may explain the underlying selectivity/promiscuity in these systems [16] that is reflected by the physiology of receptor subtypes such as the cysteinyl leukotriene receptors [17–21]. Functional interactions between GPCRs are potentially facilitated by various structures. Posttranslational modification and/or interaction between TMs [22,23] may promote the formation of dimers or higher order oligomers [24,25]; however, the *in vivo* significance of these interactions is sometimes difficult to demonstrate [24,25]. Evidence for higher order GPCR structures exists for many receptors, including the dopamine, M_3_ muscarinic, angiotensin II type 1, and the metabotropic glutamate (mGluR) receptors [26–32]. Evidence for pathogenic variants that disrupt coupling to G proteins or that disrupt receptor dimerization is emerging [33,34].

With this background in mind, we discuss the role of amino acid substitutions of accessory proteins and G proteins necessary for GPCRs signaling in human genetic disease [34–37]. Herein we review the role of variants of *GNAS* [guanine nucleotide binding protein (G protein), alpha] that encode the ubiquitous Gαs-subunit; variants of *GNB1* (GNB, G protein subunit beta) that encode the Gβ subunits and that are associated with phenotypes such as essential hypertension and obesity; and variants of *RGS4* (RGS, regulator of G protein signaling) that encode a regulator of G protein signaling associated with essential hypertension.

## G protein variants that alter GPCR signaling

Genetic variation in G protein structure resulting from imprinting, loss-of-function (LOF) or gain-of-function (GOF) variants have been reported in the genes encoding G protein subunits. We will outline the role of pathogenic and likely pathogenic variants of genes including *GNAS* and *GNB3* (encoding Gβ3), in numerous disorders. These include pseudohypoparathyroidism (PHP) [45] and melanoma [42], for which germline LOF and somatic GOF variants have been associated with disease phenotypes.

By comparison, Gγ-subunit variants implicated in human phenotypes are relatively scarce. Early studies identified that the disruption of retinal G proteins (or transducin) due to variants of alpha (α)-transducin 1 (*GNAT1*) [39] and alpha (α)-transducin 2 (*GNAT2*) [40] can result in the genetic vision deficiency, stationary night blindness. Genotype-phenotype correlations have been reported for G protein variants in a wide range of disorders. These include Albright’s hereditary osteodystrophy [37] and metabolic syndrome [38,44], including polygenic hypertension [37,43]. Other disorders [46] such as familial hypocalciuric hypercalcemia type 2 (FHH2) and autosomal dominant hypocalcemia type 2 (ADH2) [41] are associated with germline *GNA11* variants, and uveal melanoma is associated with somatic GOF variants of *GNA11* and *GNAQ* [42].

### Gβ- and Gγ-subunits

The Gβ- and Gγ-subunits, with the exception of the distinct example of Gβ5, are less diverse than Gα-subunits; however, they are integral to GPCR activation and inactivation [55–57]. In addition to their essential role in G protein activation, the Gβγ-subunits bind G protein receptor kinases, GRK2 and GRK3, but not GRK1 or GRK4-7. Membrane co-localization of GRKs and GPCRs through translocation to the membrane enables GPCR phosphorylation by GRKs associated with receptor desensitization [55–58]. Genetic variants in this system result in the complex patterns of genotype-phenotype correlation due to the diversity and tissue distribution of four or more β-subunits (Gβ1-4) in the context of their potential interaction with at least 12 γ-subunits (Gγ1-12) [32, 59–61].

While polymorphisms have been associated with a spectrum of phenotypes, variants of Gβ- and Gγ-subunits have not been identified to be pathogenic in monogenic disorders [56]. For example, a c.825C > T variant of the *GNB3* gene encoding Gβ3 may result in alternative splicing that is associated with enhanced signaling in hypertension [62].

G protein gene variants, including those of *GNB3*, have been associated with coronary artery calcification [63]. While the c.825C > T allele of *GNB3* has been associated with many phenotypes [64–72], only obesity and hypertension have been affirmed by meta-analysis [69, 71]. While the *GNB3* variant has also been associated with phenotypes such as tumor progression [73,74], drug response [75,76, and stroke [75], some studies do not find that haplotypes formed by common GNB3 polymorphisms contribute to the development of hypertension and obesity [76].

Building on this, a *de novo* p.Gly77Arg variant of *GNB2*, identified by exome sequencing, was associated with a phenotype that was similar to a pathogenic variant of its homologue, *GNB1.* The global developmental delay and hypotonia that is a feature of these patients may overlap with those manifesting disease associated with *GNB1* variants [55].

### Gα protein subunits

Gα subunit proteins can determine both the specificity of GPCR signaling and its function in generating a phenotype because there are more than 20 subunit proteins. We review observations made from selectively assaying GPCR coupling to variant or wild-type G protein subunit coupling with respect to effector proteins such as ion channels [77–79] before discussing newer tools [80–87] under ***Coupling of effector proteins by GPCRs,*** below.

**Figure 4.**
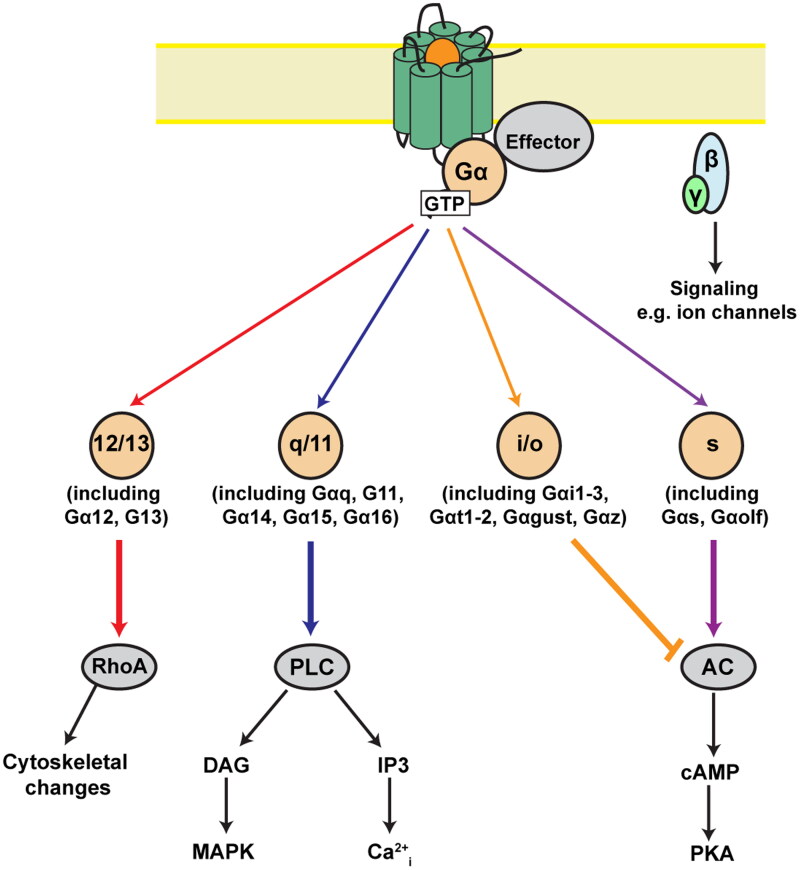
Agonist-induced conformational changes in GPCRs. Agonist-driven conformational changes within the activated GPCR allow exchange of GDP for GTP; Gα-GTP dissociates from Gβγ and stimulates downstream effectors (gray). GPCRs couple to G proteins belonging to four families. The Gα_12/13_ family activates Rho kinases to stimulate cytoskeletal rearrangements. The Gα_q/11_ family activates phospholipase C (PLC) to liberate diacylglycerol (DAG) and inositol trisphosphate (IP3) [90]. DAG activates mitogen-activated protein kinase (MAPK) cascades, while IP3 binds to IP3 receptors on the ER to release stored intracellular calcium (Ca^2+^_i_). The Gα_s_ family activates adenylate cyclase to increase cAMP which can activate a number of downstream proteins including the enzyme protein kinase A. The Gα_i/o_ family inhibits adenylate cyclase.

The rate of GTP hydrolysis varies with the type of Gα subunit expressed [16, 88] with regulators of G protein signaling (RGS) [89], a major determinant of restoring low-energy Gα-GDP [88,89]. The four Gα homologous subfamilies, Gαi/o, Gαs, Gαq/11, and Gα12/13, consisting of 20 types, have differential tissue expression (Figure 4). Exact coupling preferences and selectivities for each receptor are still being determined, with recent efforts unifying and combining available large-scale datasets toward the development of a comprehensive coupling map [16, 91].

The roles of wild-type and variant Gα subunit proteins, Gαi/o, Gαs, Gαq/11, and Gα12/13 (Figure 4) in codifying GPCR signaling, in conjunction with accessory proteins, have been well established [88,89, 91–97].

### Gα protein subunit variants

Pseudohypoparathyroidism (PHP), characterized by parathyroid hormone resistance, can result from heterozygous LOF variants of the gene encoding the Gαs subunit*, GNAS*, located at chromosome 20q13. The inheritance patterns are complicated by the fact that maternally inherited *GNAS* variants result in PHP type Ia (PHP-Ia) and paternally inherited variants result in pseudo-pseudohypoparathyroidism (PPHP) [45].

PHP-la accompanied by paradoxical testotoxicosis results from a temperature-sensitive Ala366Ser GNAS1 variant; this variant, which is constitutively active at the lower temperatures found in the testes, stimulates testosterone secretion as a consequence of constitutive adenylyl cyclase that is thermally unstable at 37 °C and results in a simultaneous PHP-1a and testotoxicosis phenotype caused by loss of G_s_ activity [92].

The *GNAS* gene is subject to alternative promoter regulation and extensive exon splicing. A number of GOF variants, including p.Arg201Leu, result in McCune-Albright’s syndrome (MAS) [98], which is characterized by fibrous dysplasia (FD) of bone, café-au-lait skin lesions, sexual precocity that is gonadotropin-independent, and nodular hyperplasia (or tumors) of pituitary somatotrophs, adrenal cortex or thyroid [99]. Similar variants have been reported to result in premature breast development and adrenocorticotropin-independent macronodular adrenal hyperplasia [100]. By contrast, heterozygous inheritance of inactivating Gαs variants in Albright’s hereditary osteodystrophy (AHO) (developmental deficits, obesity, short stature, brachydactyly, and subcutaneous ossifications) indicate that its variable phenotype results from Gαs haploinsufficiency [101–104].

Individuals who inherit *GNAS1* variants may develop AHO or autosomal dominant PPHP, depending on the parent of origin and *GNAS1* gene imprinting. Mutations derived from the father result in PPHP, which consists of predominantly skeletal defects without hormonal resistance. Individuals who inherit variants from the maternal line develop both AHO and resistance to hormones, including parathyroid hormone (PTH) [105–113], thyrotropin, growth hormone-releasing hormone, and gonadotropins [1]. When not imprinted, bi-allelic expression of both variant Gαs alleles results in the Gαs haploinsufficiency of the AHO phenotype.

Pluripotent PPH does not always result from LOF Gαs variants. In some PPHP tissues, Gs function is normal due to the role imprinting plays in determining the transcription of exon 1 of *GNAS1* in proximal tubules. Loss of GNAS maternal imprinting pattern may result from deletion of adjacent genes, *STX16* (syntaxin 16) and/or *NESP55* (SARS-CoV-2, nonstructural protein 5) [112,113]. Abnormal imprinting leads to Gαs deficiency and renal PTH resistance due to the action of repressor(s) on the Gαs promoter [108].

Recent studies of the glucagon receptor further illustrate how GPCR pharmacogenetics may be useful in identifying G protein and accessory protein functions [114]. Variants of the class B1 glucagon receptor affects cAMP (cyclic adenosine monophosphate) production and recruitment of β-arrestins. The variants that affect cAMP are associated with obesity and hypertension [114]. This study of the glucagon receptor highlight the potential for GPCR pharmacogenetics studies to identify fundamental roles for G protein and accessory proteins [114].

### Coupling of effector proteins by GPCRs

Coupling of variant and wild-type effector proteins by GPCRs [77–79] has been assessed by a wide range of tools, indicating that variations in receptor structure can change the rate at which G protein subunits are liberated [77–79, 115]. Recent studies suggest that while *GNAS* variants encoding functional changes proximal to nucleotide binding regions may share physical characteristics such as thermal melting behavior, structural properties may not align well with analogous functional changes, suggesting that unique variants may require distinct interventions [93].

Enhanced or diminished GPCR signaling can result from changes at any step in G coupling [78,79, 115]. For example, fluorescence resonance energy transfer (FRET) and bioluminescence resonance energy transfer (BRET) have permitted the analysis of ligand-binding and conformational changes within receptors, interactions between GPCRs and G protein subunits, and association with effectors and accessory proteins [80].

An open-source suite of 14 optimized BRET Gαβγ biosensors (named TRUPATH) can enable interrogation of the transducerome down to single pathway resolution within cells. The TRUPATH biosensors such as Gα15 and GαGustducin have revealed the coupling preferences for prototypic and understudied GPCRs [81]. The use of BRET to measure translocation of effectors to the plasma membrane (EMTA) has been developed. Other BRET tools determine which receptor signaling engages specific G proteins and β-arrestins. In addition, BRET has also been used to measure translocation of effectors to the plasma membrane to monitor the activation of twelve G protein subtypes. In addition to polypharmacology and biased signaling, this method can detect GPCRs that demonstrate constitutive activity and/or ligands acting as inverse agonists [82].

These technologies have allowed sensitive and dynamic monitoring of these steps. The NanoBiT system (Promega, Madison WI USA), which uses a split NanoLuc fusion of the two interacting protein partners (e.g. GPCR and β-arrestin), has allowed the quantification of β-arrestin recruitment and G protein dissociation using a single luminescent signal [83]. Such assays have recently shown how some *MC4R* variants that are associated with lower body mass index exhibit signaling bias toward β-arrestin recruitment [84]. The small size and brightness of NanoLuc means these assays are highly sensitive and NanoBiT labeling can be combined with fluorescent-tagged proteins to assess multi-protein interactions.

This has recently been used to demonstrate how WNT (secreted, lipid-modified glycoproteins) interacts with the frizzled receptor [85]. Further advances in understanding GPCR-G protein coupling have been elucidated using cell lines depleted of individual G protein families or β-arrestins by CRISPR (clustered regularly interspaced short palindromic repeats)-Cas gene editing [86]. Finally, combined computational (evolutionary and structural information) and experimental (dynamic mass distribution, internalization, and β-arrestin recruitment assay) approaches have allowed pairing between peptides and orphan-GPCRs to be elucidated, which could have a major translational impact [87].

### Accessory proteins to GPCR signaling

The diversity of genetic variants of accessory proteins in disease underscores the complexity of GPCR signaling. While accessory proteins such as β-arrestin regulate GPCR signal duration, others facilitate and focus GPCR signaling. These include receptor independent activators of G protein signaling (AGS) [88, 116,117] and RGS proteins that enhance the GTPase activity of Gα following G protein coupling [54, 89, 116–119]. G protein binding of GRK RGS domains has been demonstrated for GRK2 and GRK3, which both bind Gq in order to sequester active α-subunits away from effectors. In contrast to “classical” RGS proteins, the RGS domains of GRK do not enhance the GTPase activity of Gα. By contrast with RGS proteins that enhance Gα GTPase activity upon GPCR coupling [54, 89, 118,119], the action of AGS proteins is independent of GTPase activity [88, 116,117]. We will review the functions of AGS and RGS accessory proteins prior to discussing pathogenic variants. Selected examples of variant accessory proteins are summarized in Table 1.

**Table 1.** Selected variants in the genes encoding G protein subunits and GPCR accessory proteins.

Gene	Variant/allele	Disease/phenotype	Pharmacology	References
Gβ_3_, guanine beta-3 (*GNB3*) 12p13	825C > T SNP alternative splice	Hypertension	Shortened Gβ_3_	[65,66, 71,72, 75]
		Metabolic syndrome, obesity, insulin resistance, dyslipidemia	↑ G protein signal Abnormal stability of the functional interactions of the shortened Gβ_3_ proteins	[67–70]
		Hypertension, diabetes, obesity		[76]
		Tumor progression		[73,74]
		Polymorphic drug response marker		[120,121]
	Arg201Leu	McCune–Albright’s syndrome; fibrous dysplasia of bone; café-au- lait skin lesions; sexual precocity; pituitary, thyroid, or adrenal tumors	Activating Gαs variants with constitutive cAMP production	[98–101]
Gs, alpha (*GNAS)* 20q13.2	Insertions/deletions and SNPs, 20 % in exon 7 Haploinsufficiency	Albright’s hereditary osteodystrophy (AHO), short stature, obesity, brachydactyly, subcutaneous ossifications, developmental deficits	Inactivating Gαs variants lead to variable phenotype related to insufficient parathyroid hormone receptor (PTHR1) in chondrocytes	[102–104]
	Inheritance of paternally imprinted gene in exon 1A	Pseudopseudohypoparathyroidism (PPHP)	No renal resistance to PTH (parathyroid hormone)	[105–107]
	Ala366Ser	PHP-Ia and testotoxicosis	Thermolabile variant at 37 °C	[92]
	Inheritance of maternally imprinted gene	Pseudohypoparathyroidism type 1A (PHP)	AHO and resistance to multiple hormones	[107, 110]
Gq, alpha (*GNAQ*) 9q21	Somatic mutations in R183, Q209	Blue nevi and uveal melanoma	Increased activity of Gαq resulting in elevated MAPK responses in melanocytes	[42]
G11, alpha (*GNA11*) 19p13.3	Germline mutations in T54, L135, F220 (FHH2) and R60, G66, R181, S211, V340, F341 (ADH2)	Familial hypocalciuric hypercalcemia type 2 (FHH2) and autosomal dominant hypocalcemia (ADH2)	Inactivating mutations in FHH2 and activating mutations in ADH2 affect signaling by the calcium-sensing receptor and consequently impact upon calcium homeostasis	[41,42]
Regulator of G protein signaling 2 (*RGS2*) 1q31	1166A > C variant located in the 3′UTR	Bartter’s/Gitelman’s (BS/GS) angiotensin II-related vasomotor tones are blunted	RGS2 maximally stimulated: failure to regulate nitric oxide and cGMP	[122–125]
	Many SNPs, insertions/ deletions: 1891–1892 TC 2138–2139 AA	Bone pathophysiology Haplotypes associated with hypertension	RGS2 mRNA ↓ in fibroblasts and peripheral blood mononuclear cells	[54, 89, 119, 126]
G protein-coupled receptor kinase 1, rhodopsin kinase (*RHOK*/*GRK1*) 13q34	Exon 5 deletion	Oguchi disease, recessively inherited stationary night blindness	Impairment of GRK1- mediated desensitization of rhodopsin	[127–130]
G protein-coupled receptor kinase 4 (*GRK4*)	Arg65Leu, Ala142Val, and Ala486Vval	Hypertension, sodium sensitivity	GRK4 activity increased: ↑ Dopamine D_1_ receptor desensitization ↑ Angiotensin II type 1 receptor expression	[131–134]
Regulator of G protein signaling 10 (*RGS10*)	c.489_491del:p.E163del and c.G511T:p.A171S	Short stature	Mislocalization of RGS10 Reduced chemokine signaling Reduced hormone signaling	[135]

cAMP, cyclic adenosine monophosphate; cGMP, cyclic guanosine monophosphate; mRNA, messenger RNA; SNP, single-nucleotide polymorphism.

Originally identified using a yeast-two hybrid screen, there are at least ten structurally diverse AGSs that activate Gβγ-dependent signaling [126]. AGS protein classification is as follows: class I, which function as guanine nucleotide exchange factors (GEFs) that can be used to promote dissociation of heterotrimers, resulting in GαGTP and free Gβγ; class II, which act as guanosine dissociation inhibitors (GDIs) that expel free Gβγ; and class III, which interact with either Gβγ or heterotrimer and promote heterotrimer dissociation that is nucleotide exchange independent [88]. Each class has its own selectivity and tissue expression: class I consists of AGS1, R1C8A, RiC8B, and GIV; class II, the largest subgroup, consists of AGS3, AGS4, AGS5, and AGS6 [136]; and class III consists of AGS2, AGS7, and AGS8 [88].

By contrast, inactivation of the signal associated with G coupling of GPCRs is facilitated by RGS proteins because they are GTPase accelerating proteins (GAPs) and result in hydrolysis of Gα-bound GTP to GDP. The deactivation of Gα subunits is dependent on the extent and timing of RGS protein action [54, 89, 118,119].

The selectivity of RGS proteins for the complete set of Gα substrates has been examined using real-time kinetic measurements in living cells. This suggests that these proteins are selectively bar-coded for G protein recognition and swapping bar codes can switch G protein preferences. As a result, coding variants in these proteins can result in functional changes associated with specific phenotypes. In the context of the decoded signal selectivity, this serves to identify the influence of molecular recognition principles of this class of proteins on many phenotypes [137].

There are approximately twenty genes encoding RGS proteins from which isoforms are generated from splice variants [89]. The RGS proteins interact with 16 Gα genes [137] to create a complex pattern of regulation [122–125, 136–152]. The classification of RGS proteins as R4, RZ, R7 and R12 is based on sequence conservation unrelated to the RGS domain itself. The R4 subfamily, characterized by an amino-terminal α-helix, is the largest subgroup. The RZ group, characterized by an amino-terminal cysteine (Cys) domain, consists of RGS17, RGS19, and RGS20; this group is characterized by numerous features that include Disheveled, Egl-10 and Pleckstrin (DEP) domains. The R12 family is characterized by numerous features that include Ras-binding domains (RBDs) [138].

Because there are many RGS proteins and Gα subunit species, great diversity of inactivation is possible. While most RGS proteins promote GTP hydrolysis by the Gαi subfamily [138], some act at Gαq [142]. Similarly, some effectors, such as phospholipase Cβ (PLCβ) [142] and leukemia-associated RhoGEF (LARG), can act at Gα12/13 proteins as GAPs [54, 138, 147] in a process known as “effector antagonism” [89].

The evidence suggests that many RGS proteins can bind directly to GPCRs without any recruitment; however, Gα subunits (Gαs) or linked GPCRs expedite the process [138]. Although activated Gα tends to have a high affinity for RGS proteins, this is not always necessary, and RGS binding to phospholipids may also play a role. However, scaffold proteins such as spinophilin and GPCR effectors may facilitate recruitment in some cases [89]. RGS proteins are therefore selectively sorted at the plasma membrane, a process that can optimize GAP activity that quenches the signal [89, 136–138].

## RGS proteins implicated in disease

RGS proteins participate in many disease processes, including cancer, cardiovascular disease, and disorders of the central nervous system [138]. They have been implicated in variable response to opioids and antipsychotics and in the development of Parkinson’s disease (PD). RGS9 is a critical component of the phototransduction in retinal neurons. Loss of RGS9 results in a visual disorder in which patients cannot adapt to changes in light, while loss of RGS2 amplifies signaling by angiotensin AT1 receptor that may contribute to hypertension and cardiac remodeling (also regulated by RGS14) [139].

Interestingly, feedback loops such as the AT1 receptor feedback loop that regulates the expression of RGS2, RGS10, and RGS14 have been reported. Changes in RGS expression contribute directly to disease initiation, disease progression, treatment efficacy, tolerance, and unwanted side effects [138]. Rational drug discovery for hypertension may optimize RGS signaling [122, 125, 139, 147, 149,150, 152]. Likely pathogenic RGS variants may disrupt their interaction with the receptor to impair normal deactivation of signaling [54, 119, 122–126, 135–152].

### Polymorphisms of the RGS gene in hypertension

While variants such as c.1166>C variant located in the 3′ untranslated region (3′UTR) of angiotensin 1 receptor have been implicated in Bartter’s/Gitelman’s syndrome patients [122,123], RGS2 is also implicated. Although the frequencies of *RGS2* gene variants are often low, the more common c.1114C > G was reported to be associated with hypertension and with lower *RGS2* gene expression in some populations [124]. The C1114G polymorphism was associated with differential RGS2 expression, with the lowest values in hypertensives who were homozygous for the variant allele. RGS2 mRNA expression was significantly lower in peripheral blood mononuclear cells (PBMCs) and in fibroblasts from hypertensive patients. These findings have led to the hypothesis that insufficient RGS2 expression may result in a failure to terminate signaling by RGS activation of the Gα subunit’s GTPase [54, 89, 119, 126].

In addition to the single nucleotide variants (SNVs) of *RGS2*, intronic 1891 to 1892 TC and 2138 to 2139 AA in/del variants of *RGS2* are relatively common and have been reported to be in linkage disequilibrium. Among African-Americans, two haplotypes have been reported to have significantly different frequencies between hypertensives and normotensives. The intronic in/del haplotypes may serve as ethnicity-specific genetic variants for essential hypertension, which may reflect the unique epidemiology of hypertension in this population [125].

### RGS10 variants and short stature

A clinical and molecular phenotype presenting with short stature and immunodeficiency is associated with *RGS10* variants Glu163del and p.Ala171Ser [135]. In addition to short stature due to growth hormone deficiency, this global phenotype includes hypergammaglobulinemia, lymphatic abnormalities and recurrent infections [135]. While the activity of the RGS10 variants is retained, PKA-mediated phosphorylation is aberrant, and the protein distribution differs from wild-type. Mis-localization of the RGS10 protein results in attenuated chemokine signaling [135].

## G protein receptor kinases (GRK) and human phenotypes

While agonist exposure of a GPCR to an agonist normally produces self-limited activation [2,39,64,153], disease states may often be associated with constitutive activation. This can result from failure of receptor inactivation in response to agonist (Figure 3). Inactivation is the process that follows phosphorylation of a specific combination of GPCR residues that constitute bar codes [153], which are unique to different GRKs. Different GRKs may phosphorylate different residues (i.e. GRKs 2 and 3 phosphorylate different residues than GRKs 5 and 6), patterns of phosphorylation that have been referred to as a “bar code”. The “bar code” of a GPCR may depend not only on the GRK but also on the agonist that has been used to stimulate the GPCR. This has, for example, been convincingly demonstrated for the mu-opioid receptor. GRK phosphorylation often does not alter G protein interaction; the receptor is desensitized only after arrestin binding, which in turn inhibits second-messenger production such as cAMP, in response to the agonist exposure. We will briefly review interrelated processes involved in GPCR inactivation, i.e. desensitization, internalization, and receptor trafficking, before discussing pathogenic variants of GPCR kinase (GRK) in disease states [16].

**Figure 5.**
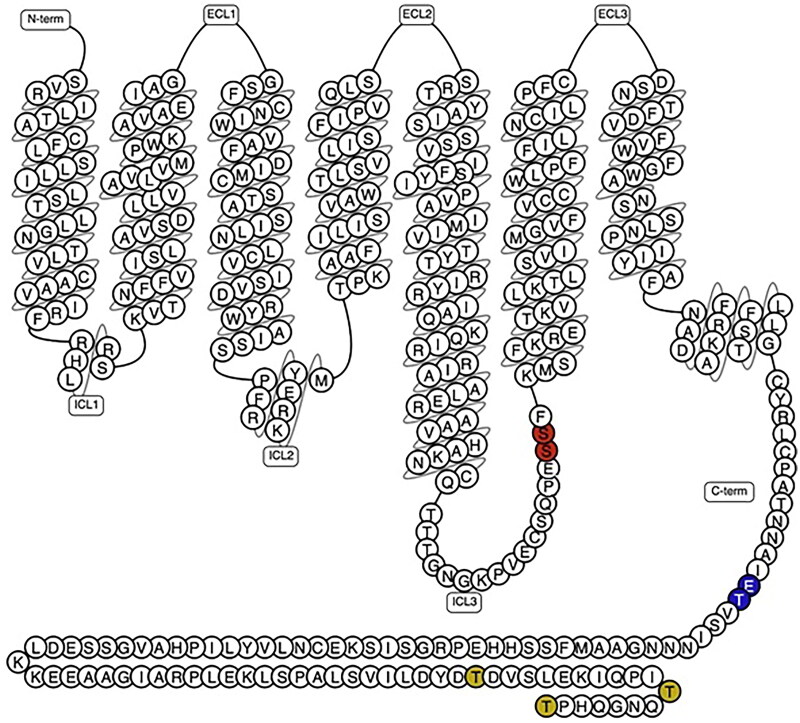
Amino acid residues required for receptor desensitization and internalization: the dopamine D1 receptor study in Chinese hamster ovary (CHO) cells. The substitution of 256Ser and 257Ser to Ala has been reported to be critical to arrestin-mediated desensitization in HEK293 cells [157]. By contrast, substitution of 359Glu or 360Thr with Ala in CHO cells has been reported to result in desensitization-deficient mutants of the dopamine D1 receptor that are still able to internalize to some extent [153]. Phosphorylation sites in a 12-amino acid stretch of the distal carboxyl tail (428Thr to 439Thr and 446Thr) may be involved in internalization of the receptor in HEK 293 cels [153]. Some studies suggest that the residues in this region of the carboxyl tail may interact with the third loop residues to modulate these effects [158] and that this region may be involved in endocytosis [159].

Classical desensitization takes place within a time frame of seconds to minutes. Agonist dependent homologous desensitization follows agonist exposure [2]. The resulting conformational changes secondary to GRK activity can be dependent on specific sequences of amino acid residues, or barcodes [58, 154–156], as shown in Figure 5. Originally observed to regulate rhodopsin, homologous desensitization in GPCRs is modeled to rapidly follow agonist dependent GRK phosphorylation [31, 160,161].

By contrast, heterologous desensitization is any desensitization that does not depend on the presence of agonist. It takes place over a longer time course in response to protein kinase C (or another kinase such as protein kinase A or casein kinase) activity (Figure 4) [162,163]. For example, beta-adrenoceptor stimulation may result in protein kinase A activation, which in turn may phosphorylate and potentially desensitize other receptors [31, 160–164].

**Figure 6.**
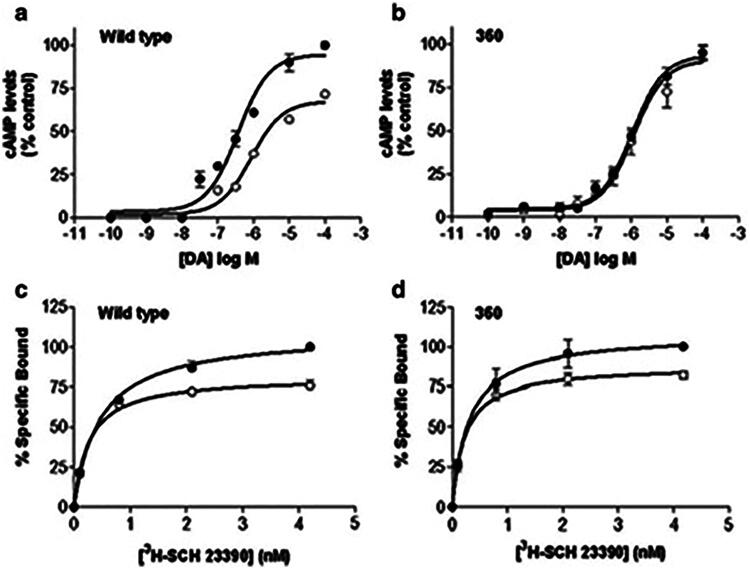
*In vitro* effects of mutation on desensitization and internalization of the dopamine D1 receptor. Dose-dependent intracellular cAMP accumulation (a and b) and binding curves (c and d) for artificial ligand (SCH 23390) are shown for three constructs: control (wild-type, a and c) and the Thr360Ala mutant (360, b and d). In the desensitization experiments, CHO cells were preincubated with 10 μmol/L dopamine (open circle) or vehicle (closed circle) for 20 min and increasing concentrations of dopamine (10^−10^ to 10^−4 ^μmol/L) were added to assess cAMP accumulation. Desensitization of the wild-type receptor (a), defined by an increase in Km and decrease in Vmax for agonist-pretreated compared with naïve cells, was abolished (with respect to efficacy and potency) for Thr360Ala (b). Conversely, internalization, defined as a loss of cell surface receptors (measured by decreased maximal binding or Bmax assessed by SCH23390 binding) is unchanged from wild-type (c) after pretreatment with 10 μmol/L dopamine (open circle, compared to vehicle (closed circle), for the Thr360Ala mutation (d) [153].

Clusters of serine or threonine residues in the third intracellular loop and/or carboxyl tail are required for homologous desensitization of many receptors [165–169]. GRK2-, GRK3- and GRK5-mediated phosphorylation has been implicated in homologous desensitization of many receptors [153,170]. For some receptors [166,167,171–179], including the dopamine D1 receptor [153], the motifs may be located in the carboxyl tail or third intracellular loop (IC3), as shown in Figure 6. This may reflect the role of these structures in trafficking of desensitized receptors [159,180] secondary to conformational changes in receptor structure [157, 181].

Intermediaries such as arrestins promote the activity of effectors as a result of second messenger cascades in the context of a variety of other partners. For example, in HEK293 cells, dopamine D1 receptor binding to G proteins instead of arrestin may take place in the context of preferential activation of ERK1 (ERK, extracellular signal-regulated kinase) or ERK2 following receptor phosphorylation. Dynamic functional outcomes resulting from GPCR signaling biases [181] differ among receptor systems [158].

GRK-dependent recruitment of β-arrestin may also initiate internalization [182], as shown in Figure 3. Serine and threonine residues in the carboxyl tail are involved in both desensitization and internalization of the dopamine D1 [153] (Figure 5) and β_2_-adrenergic receptors [165,183–185]. Internalization, therefore, may follow desensitization [186–188] or it may occur independently [189] with or without the influence of other regulatory processes [190]. While β-arrestin has been shown to be involved with the internalization of over 30 GPCRs [127, 191–203], various non-arrestin mechanisms of internalization may act in some cases [204–208].

### The GRK family of enzymes

Because genetic variants of GRK enzymes result in disease [127], the GRK family of enzymes is important [127, 209]. GRK1, rhodopsin kinase, was the first enzyme described [210] in what would become, with GRK7, the visual GRK subfamily. GRK2, originally purified from cells as β adrenergic receptor kinase (βARK) (205) and later shown to be homologous to rhodopsin kinase [211], and GRK3 comprise the beta-gamma GRK subfamily. GRK4, 5 and 6 comprise a distinct subfamily [210,211]. Figure 2 illustrates the role of the GRKs in GPCR signaling. GRKs can be distinguished by (1) the structural homology; (2) amino acid “bar code” phosphorylated; (3) the kinetics of each enzyme [127, 209]; and (4) deep-phenotyping associated with GRK dysregulation. GRKs are relevant to variant GPCRs for many reasons, including the fact that GOF mutations in GPCRs are frequently constitutively phosphorylated. By contrast, GPCRs with LOF variants may result in inadequate receptor desensitization and sequestration [127, 209,210].

The GRK2 subfamily, consisting of GRK2 and GRK3, acts on a wide range of GPCRs that are expressed in many tissues. Of all the GRK family, the GRK2 amino acid sequence is most widely divergent from GRK1, which may also be a factor in defining which tissues are affected by ectopic GPCR phosphorylation [180]. However, substrate specificity is also defined by the amino acid sequence of GPCRs adjacent to serine/threonine residues. A significant factor in determining which tissues are affected by pathogenic GPCR mutations may be substrate specificity of the GRKs acting on the receptors [210]. GRK2 enzymes contribute to disease. For example, GRK2 GOF variants affect the luteinizing hormone receptors (LHR) that are associated with Leydig cell hyperplasia [212].

By contrast, GRK4, including the α/β- and γ/δ-isoforms, specifically phosphorylates sites adjacent to basic amino acid residues. The GRK4 subfamily consists of the GRK4, GRK5, and GRK6 enzymes [58]. GRK4 may play a role in the dopamine D_1_ receptor and angiotensin 1 receptor desensitization [131] in the context of genetic and acquired hypertension [131,132]. GRK4 is relevant for the function of cilia in eukaryotic cells [133].

### GRK1 and Oguchi disease

The pathogenic variants of the prototypical rhodopsin kinase underlie several inherited retinal disorders, including Oguchi disease [127,] a rare, recessively inherited retinopathy [128]. Pathogenic variants such as a deletion of exon 5 of the *GRK1* gene result in a null mutation [128]. Both homozygous and heterozygous states for this mutation lead to disease [129,130]. A dominant negative effect or a GRK gene dose effect may be involved.

GRK1 null variants result in disruption of the pathway of light-dependent rhodopsin phosphorylation and arrestin-mediated quenching of signal transduction in photoreceptor cells. This results in rhodopsin remaining in the activated state even in the absence of ligand such as, in the case of retinopathies, light.

Disruptions in rhodopsin signaling result in alterations in the phosphorylation of rhodopsin by GRK1, the specialized GRK enzyme expressed in the retina that is largely responsible for rapidly desensitizing rhodopsin when it is exposed to light. These GRK1 pathogenic variants result in impaired rhodopsin desensitization, a process that normally quenches light-induced signal transduction in photoreceptor cells. GRK1 deficiency cannot be compensated for by GRK7 because GRK1 is expressed mostly in rods whereas GRK7 is expressed in cones [58].

### GRK4 isoforms and essential hypertension

The p.Arg65Leu, p.Ala142Val, and p.Ala486Val variants of the γ-isoform of GRK4 expressed in the kidneys are implicated by genetic functional studies in increased phosphorylation by GRK enzymes and reduced receptor-mediated cAMP phosphorylation [131,132]. GRK4 variants associated with inappropriate desensitization of the dopamine D1 receptor in renal proximal tubules in hypertension may result in the decreased ability of the kidney to eliminate a sodium chloride load, a key risk factor in the development of hypertension. This suggests that dysregulation of GPCR systems might be corrected by blocking the effects of GRK4 in patients who harbor GRK4 polymorphisms. The principle of targeting accessory proteins might be applicable to other disorders that involve disruptions to normal GPCR signaling [131, 133].

The etiology of essential hypertension is best understood in the context of the way in which pathogenic GRK4 variants interact with other systems involved in blood pressure regulation. In this context, GRK4 variants contribute to hypertension through disruption of the signaling of dopamine D1 receptor and angiotensin type 1 receptor (AT1R). Expression of variants of the GRK4 γ-isoform in mice has been reported to result in hypertension because of both impaired dopamine D1 receptor function and increased expression and activity of the AT1R due to phosphorylation of histone deacetylase type 1 (HD1). This effect is magnified because increased AT1R expression itself increases the nuclear export of HD1 to the cytoplasm, further increasing AT1R expression and heightened pressor response to angiotensin II. These findings were confirmed in mice by the discovery that hypertension was normalized with both AT1R blockade and the deletion of the GRK4 γ-isoform [134]. Based on these data, there may be potential for rational drug design for hypertension predicated on targeting receptors with opposite physiology. Thus, GRKs such as GRK4 may be therapeutic targets in hypertension [132].

## Conclusions

We have reviewed the role of genetic variants in G protein subunits and accessory protein in prototypical disorders. These include pathogenic variants of G protein subunits such as the *GNB3* gene in hypertension, activating *GNAS* variants in McCune-Albright’s syndrome [98], heterozygous inheritance of inactivating *GNAS* variants in AHO [101–104], and PPH resulting from heterozygous LOF *GNAS* variants [45].

Disruption of genes encoding RGS proteins have been reported in human phenotypes. Inactivating *RGS9* variants result in changes in light adaptation. LOF variants of *RGS2* may contribute to hypertension [139] due to their role in regulating AT1R activity and signaling, including in Bartter’s/Gitelman’s syndrome [122,123].

Variants of several genes encoding GRK enzymes have been identified since the role of *GRK1* variants in Oguchi disease was identified. Variants of *GRK4* have been implicated in the development of hypertension [131,132]. The role of G proteins and accessory proteins in phenotype expressivity may be predicted from signaling bias. For example, functional studies of a dopamine D2 (*DRD2*) p. Ile 212Phe variant in a hyperkinetic disorder identified evidence for signaling bias. The pathogenic variant had higher basal activity coupled to Gα_oA_ than Gα_i1_. GRK2 activity is necessary for the phenotypic expression. While DRD2 p.Ile212Phe interacts with higher potency at the Gαi1β1γ2 heterotrimer, resulting in slightly elevated basal Gαi/o activation, this is dependent upon signaling bias determined by GRK2 expression [213].

**Figure 7.**
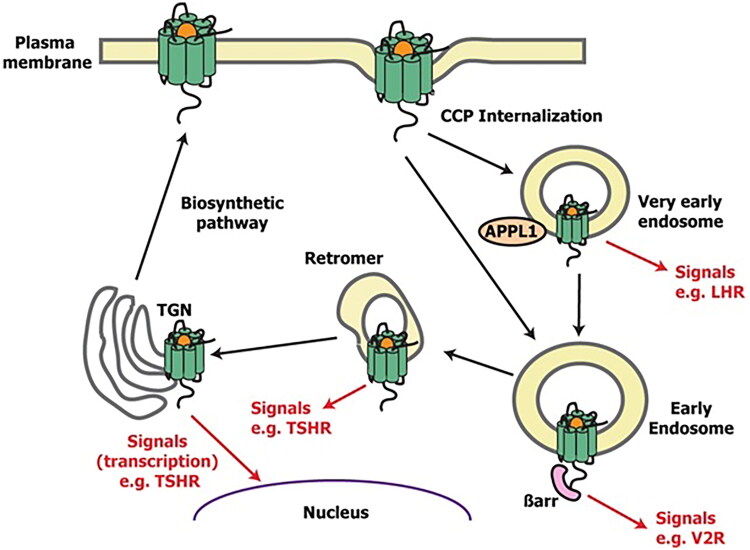
Schematic showing intracellular sites at which GPCR signaling has been described. GPCRs are produced within the biosynthetic pathway and reside in plasma membranes from which they generate G protein signaling. Following receptor internalization *via* clathrin-coated pits (CCPs) GPCRs can internalize to very early endosomes (VEEs, which are characterized by the presence of leucine zipper 1), or early endosomes, from which additional G protein signaling can be produced. GPCRs including LHR and FFAR2 can signal from VEEs, while receptors including V2R and PTH1R can signal from early endosomes, which may involve β-arrestin (βarr). GPCRs may be trafficked *via* retromer complexes to the trans-Golgi network. TSHR signals from these sites to generate cAMP and CREB-mediated transcription.

Appreciation of the role of accessory proteins in phenotype expression is supported by data suggesting that β-arrestin recruitment and internalization may not always terminate signaling. The role of accessory protein variants can be interpreted in the context that some GPCRs are able to persistently stimulate or re-activate G proteins at endosomal compartments or within the biosynthetic pathway [195, 214] (Figure 7). A growing list of receptors has now been shown to stimulate G protein signaling from intracellular compartments including the receptors for thyrotropin, parathyroid hormone (PTH1R), luteinizing hormone (LHR), GLP-1 (GLP1R), arginine vasopressin (V2R), and some opioids (δ-OR).

The integrity of accessory protein mechanisms can be seen to be critical to sustained signaling. Some GPCRs, such as the V2R, can simultaneously bind G protein and β-arrestin to activate signaling from endosomes [215]. These complexes serve as a scaffold to recruit signaling molecules and retromer components that can switch off endosomal signaling by some GPCRs [215]. Other receptors can signal from very early endosomes (VEEs) that involve the adaptor protein, phosphotyrosine, interacting with PH (pleckstrin homology) domain and leucine zipper 1 adaptor protein to activate distinct signal pathways. Such signaling has been shown for the free-fatty acid receptor-2 (FFAR2) to regulate propionate-induced GLP-1 release, and for LHR to activate a pathway which that may influence reproduction and pregnancy [216,217]. Several GPCRs signal from the trans-Golgi network, either by retrograde trafficking from endosomes (e.g. TSHR) or from the synthetic pathway (e.g. β1AR) [217]. At the trans-Golgi network, TSHR can activate CREB (cAMP response element-binding protein)-dependent gene transcription due to its close proximity to the nucleus [218].

Targeting endosomal signaling is also emerging as a new therapeutic strategy. The innate propensity of PTH to activate sustained signaling from endosomes compared to the short-lived signals from the plasma membrane by PTHrP, have been manipulated to design long-acting PTH, which triggers substantially longer cAMP responses and prolonged hypercalcemia [219]. Finally, the loss of sustained signaling by the PTH1R and CaSR have been associated with hypo- and hypercalcemia, respectively [220,221], further demonstrating the importance of such signaling in physiology.

Studying the wider impact of genetic variants of genes encoding accessory proteins on intracellular GPCR signaling has been hampered by the nature of experimental approaches to investigate these phenomena. These can involve chemical manipulation such as endocytic blockade by small molecules (e.g. by Dyngo-4a, an inhibitor of clathrin-mediated endocytosis), which can have adverse effects on other components of cell signaling, while other studies that investigate signaling following agonist wash-out may not achieve complete agonist removal [214]. Recently developed methods such as the use of nanobodies to localize active-conformation GPCRs to endosomes, may be able to assess the impact of these variants on the refractory period in these processes [214].

The developments discussed in the present article will enhance our understanding of the impact of G protein and accessory protein variants on fundamental mechanisms involved in GPCR synthesis, transport to the membrane, ligand binding, and activation and inactivation G protein subunits [90, 222]. In the second article in the series, we build upon this background to discuss “G protein-coupled receptor (GPCR) gene variants and human genetic disease” [223] and in the third article, we survey “G protein-coupled receptor (GPCR) pharmacogenomics” [224].

## Abbreviations

AHO: Albright’s hereditary osteodystrophy
cAMP: cyclic adenosine monophosphate
CaSR: calcium sensing receptor
CREB: cAMP response element-binding protein
CRISPR: clustered regularly interspaced short palindromic repeats
ECL: extracellular loop
FFAR2: free-fatty acid receptor-2
GNAS: guanine nucleotide binding protein (G protein), alpha
GNB: G protein subunit beta
GOF: gain-of-function
GPCR: G protein-coupled receptor
LHR: luteinizing hormone receptor
LOF: loss-of-function
PHP: pseudohypoparathyroidism
PPHP: pseudo-pseudohypoparathyroidism
PTH: parathyroid hormone
RGS: regulator of G protein signaling
TM: transmembrane domain
V2R: arginine vasopressin
VEE: very early endosome
